# Severe Acute Respiratory Syndrome: Temporal Stability and Geographic Variation in Death Rates and Doubling Times

**DOI:** 10.3201/eid0908.030334

**Published:** 2003-08

**Authors:** Alison P. Galvani, Xiudong Lei, Nicholas P. Jewell

**Affiliations:** * University of California, Berkeley, California, USA

## Abstract

We analyzed temporal stability and geographic trends in cumulative case-fatality rates and average doubling times of severe acute respiratory syndrome (SARS). In part, we account for correlations between case-fatality rates and doubling times through differences in control measures. Factors that may alter future estimates of case-fatality rates, reasons for heterogeneity in doubling times among countries, and implications for the control of SARS are discussed.

Concern over the emergence of severe acute respiratory syndrome (SARS) has persisted as epidemic continues in the first months of 2003, despite control efforts. As of May 12, 2003, the World Health Organization (WHO) had reported 7,447 cases with 552 deaths in >30 countries. The most affected locations are China, Hong Kong, Singapore, Viet Nam, Taiwan, and Canada. (Our study focuses on Canada.) Doubling times (i.e., the period required for the number of cases in the epidemic to double) and case-fatality rates (CFRs) are fundamental to the epidemiology and potential public health impact of SARS. Doubling times are a measure of the rate of spread of disease and also indicate the magnitude of control efforts required to curtail spread. Because doubling times change substantially over the course of an epidemic, current estimates should not be used to extrapolate into the future.

CFRs of SARS have typically been estimated by dividing the number of deaths by the total number of cases. This method is sufficient for an advanced epidemic. However, the method is not accurate at an early stage of an epidemic, particularly when the time from infection to recovery or death is not brief, relative to the duration of the epidemic, as is currently true with SARS. The method underestimates the CFR because it does not account for a proportion of currently infected persons’ dying from the disease. A more accurate method would be to divide the number of deaths by the total number of deaths plus the number of persons who recovered. By applying this method to publicly available WHO data (*1*), the cumulative CFR estimates appear more stable, relatively constant within a country (aside from Taiwan, which is in the earliest stage of its epidemic) but varying considerably among countries (Figure 1).

**Figure 1:**
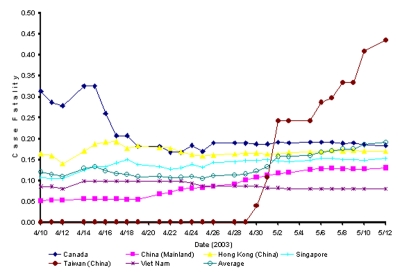
Cumulative case-fatality rates for severe acute respiratory syndrome over time.

Average overall CFR for all countries increased from 10.4% on April 21 to 14.7% on May 12 (largely attributable to the sudden rise in CFRs in China and Taiwan). In countries with few deaths, this estimate of CFR may be a slight overestimate if the time from infection to death tends to be shorter than time to recovery. However, recent cohort data from Hong Kong (*2*) suggest the opposite, implying that our crude estimates of CFR may still be underestimates. Such inaccuracies are, however, unlikely to modify the results of a general comparison of CFRs across countries. Nevertheless, caution is warranted in comparing CFRs across countries since differences may exist in the various surveillance systems that report cases and the number of persons who recovered.

Figure 1 does not directly provide information on whether the CFR shows temporal trends in any country as it plots the average CFR since the beginning of the epidemic. Unfortunately, the publicly available WHO data do not permit a CFR to be estimated over time since cases reported in one period are not linked to recoveries at the same or future time. The determination of factors, including date of infection, that influence death rates awaits detailed analyses of cohort data on infected persons.

We identify an inverse relationship between the average CFR and the average doubling time for different countries (Figure 2). (The average doubling time is a cumulative measure reflecting average growth from the beginning of reported data and is estimated by the length of a time period divided by the log_2_ of the relative growth in numbers of reported cases during the same period; see Appendix.) This relationship is probably generated by the influence of the efficacy of control policy affecting both parameters, rather than a reflection of different characteristics of viral infectiousness and virulence across epidemics. The rapid hospitalization of infectious persons is likely to reduce the CFR and increase the doubling time (by reducing the spread of SARS). Consistent with this explanation is the successful containment of a sizable epidemic in Viet Nam and the relatively low CFR and long doubling time there. In contrast, Canada has the highest CFR and shortest doubling time (except for Taiwan, where the CFR has yet to reach a steady state). Stochasticity in personal contacts plays a key role during the invasion phase of the epidemic. In Toronto, the stochasticity of social contacts resulted in a second outbreak after public health officials thought that SARS had been controlled. Transmission also occurred in Toronto before public awareness of SARS was widespread, resulting in delayed hospitalization of the first few patients; this scenario, in turn, facilitated transmission and may have elevated death rates (*3*). Thus, the means of disease introduction may be important in determining early doubling times and CFR values. These factors, combined with small sample sizes, may have been the cause of particularly high CFRs from April 10 to April 15 in Canada.

**Figure 2:**
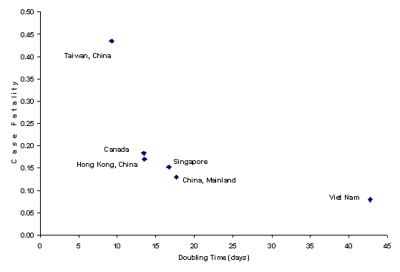
Cumulative case-fatality rate for severe acute respiratory syndrome compared with average doubling time as of May 12, 2003.

Variation in CFRs among countries will arise from differences in intensity and speed of medical care, age structure of the population (older infected patients are more likely to die [*2*]), and factors such as coinfection. For example, the high prevalence of coinfection with other respiratory diseases, such as infections caused by *Chlamydia pneumoniae* (*4*), *C. psittaci,* and paramyxoviruses in China, could increase the CFR there. Likewise, should SARS spread in Africa, the disease could have a devastating effect, given its high prevalence of tuberculosis and HIV/AIDS.

Estimates of CFR may change as polymerase chain reaction (PCR) assays become more widely used in diagnosis (*5*). Diagnostic tests could identify mild cases that currently are not reported. Our estimate of the size of the epidemic would then increase in terms of number of cases, but the estimates of CFR would decrease. Conversely, PCR tests might eliminate the diagnosis of SARS in some suspected cases. Ultimately, accurate estimates of population distributions of parameters reflecting the clinical course of disease will be best provided by follow-up of clearly defined cohorts of infected persons identified by appropriate diagnostic procedures.

As an epidemic declines, the doubling time increases. Variation in doubling time among countries probably arises from variation in both transmission rates and control efforts (Figure 3). Transmission rate (with units of time^-1^) is determined by the expected number of susceptible persons with whom each infectious person comes into contact during a time unit in their infectious periods and by the probability of disease transmission per contact. High-density population centers, crowded public transportation systems, and hospital waiting rooms increase the number of contacts, while personal hygiene affects the probability that transmission will occur with each contact. In all countries, seasonal effects may also play a substantial role with the virus spreading faster in winter.

**Figure 3:**
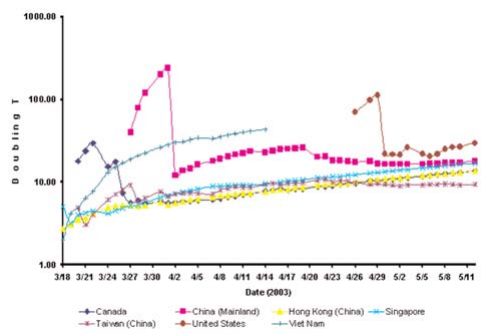
Time series of log of average doubling time for severe acute respiratory syndrome.

In Viet Nam, the doubling time increased over the period that the epidemic was being controled (Figure 3). The dramatic drop in doubling time in China in early April corresponds to a change in reporting practices (Figure 3). Similarly, in the United States, a shift in the definition of SARS to correspond to that recommended by WHO complicates estimation of doubling time. However, the doubling time appears to be relatively long in the United States because most cases are caused by seeding from travel to Asia, with few cases occurring from local transmission.

Epidemic models may provide a framework for evaluating alternative control measures. Central to the accurate parameterization of epidemic models is the reproductive ratio, *R*_0_, which is the average number of secondary cases generated by one initial infection in a susceptible population in the absence of control measures (*6*). *R*_0_ defines a threshold that determines whether an infection is likely to spread. If *R*_0_ is <1, each infection will not replace itself, on average, and the disease will likely die out, although in such cases spatial dynamics, latency, and stochastic variation may contribute to localized flare-ups of the disease that may persist for a long time. Thus, *R*_0_ also defines the level of intervention required to contain an epidemic. The doubling rate can be used to calculate *R*_0,_ given that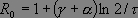, where *γ* is the duration of the incubating period, *α* is the duration of the symptomatic period, and *τ* is the doubling time (*7*). Accurate characterization of the incubation and symptomatic periods is essential to the translation of doubling times to *R*_0_. Typical estimates of the incubation period for SARS range from approximately 2 to 10 days (median and mean 5 days) (*4*,*8*), whereas the symptomatic period has a mean (± standard deviation) of 16±8 days (*4*,*8*). Recent data from Hong Kong (*2*) suggest somewhat longer incubation on average. However, severe infections may be overrepresented in current estimates, which have been based largely on persons who have received intensive medical treatment, another factor that may affect the symptomatic period. At this point in any of the epidemics, we are reluctant to use this approach for calculating *R*_0_ from doubling times since the latter is confounded by evolving control policies (e.g., Hong Kong, Toronto); the most natural epidemic (in Guangdong Province) offers the least complete data.

Figure 4 plots the reported case counts in China, together with an exponential curve fitted to a smooth version of the counts (to allow for the discreteness in reports in early April). The estimated doubling time from this curve is 16.2 (which closely matches the May 3 value for China in Figure 3 of 16.3; the May 12 doubling time is now 17.7, since the growth in counts has declined in the 9 days after May 3). The curve suggests that 502 cases existed in China on March 17 (with a 95% confidence interval of 468 to 538) (Appendix), which is consistent with underreporting at that time. Control measures, evolving contact patterns, stochastic effects, and potential acquired immunity will all impact this doubling time (equivalently, the growth in case counts) and ultimately lead to a flattening of the growth observed to date. This decline in the rate of growth can be seen already in the last week of data from China, although whether this decline is real or due to delayed reporting is not yet known.

**Figure 4:**
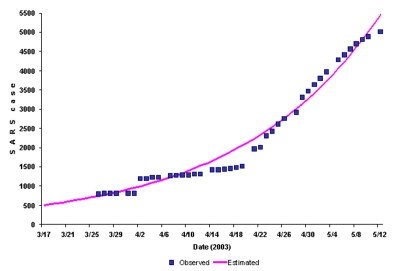
Observed and expected cumulative number of cases of severe acute respiratory syndrome in China.

The rapid increase in the number of cases in China suggests an urgent need to control the epidemic in Asia before it gains further momentum. Containment of an outbreak at an early stage affords a greater chance of success than does a later response and clearly puts less strain on the healthcare system. Isolation of cases, infection-control measures in hospitals, and vigilant surveillance at community and population levels are imperative. Failing this, SARS could become endemic in China, particularly if it evolves antigenically to evade pre-existing immunity, such that recovered patients could be reinfected, as is the case for influenza (*9*). In this eventuality, international travel would continually seed new cases in other parts of the world. SARS reaffirms what we have previously learned from other infectious diseases, namely that epidemic control is a global concern and not the problem of one or a few nations.

## Supplementary Material

Appendix Severe Acute Respiratory Syndrome: Temporal Stability and Geographic Variation in Case-Fatality Rates and Doubling Times

## References

[1] Cumulative number of reported cases of severe acute respiratory syndrome (SARS). Geneva: World Health Organization, 2003. Cited May 12, 2003. Available from: URL: http://www.who.int/csr/sarscountry/en/

[2] Donnelly CA, Ghani AC, Leung GM, Hedley AJ, Fraser C, Riley S, Epidemiological determinants of spread of causal agent of severe acute respiratory syndrome in Hong Kong. Lancet online May 7, 2003. Available from: URL: http://image.thelancet.com/extras/03art4453web.pdf 10.1016/S0140-6736(03)13410-1 PMC7112380 12781533

[3] Cleveland WS. Robust locally weighted regression and smoothing scatterplots. J Am Stat Assoc. 1979;74:829–36. 10.2307/2286407

[4] Poutanen SM, Low DE, Henry B, Finkelstein S, Rose D, Green K, Identification of severe acute respiratory syndrome in Canada. N Engl J Med. 2003;348:1995–2005. 10.1056/NEJMoa030634 12671061

[5] Koh WP, Taylor MB, Hughes K, Chew SK, Fong CW, Phoon MC, Seroprevalence of IgG antibodies against *Chlamydia pneumoniae* in Chinese, Malays and Asian Indians in Singapore. Int J Epidemiol. 2002;31:1001–7. 10.1093/ije/31.5.1001 12435775

[6] Dorsten C, Gunther S, Preiser W, Werf S, Brodt HR, Becker S, Identification of a novel coronavirus in patients with severe acute respiratory syndrome. N Engl J Med. 2003;348:1967–76. 10.1056/NEJMoa030747 12690091

[7] Anderson RM, May RM. Infectious diseases of humans. Oxford (UK): Oxford University Press; 1991.

[8] Tsang KW, Ho PL, Ooi GC, Yee WK, Wang T, Chan-Yueng M, A cluster of severe acute respiratory syndrome in Hong Kong. N Engl J Med. 2003;348:1977–85. 10.1056/NEJMoa030666 12671062

[9] Ferguson NM, Galvani AP, Bush RM. Ecological and immunological determinants of influenza evolution. Nature. 2003;422:428–33. 10.1038/nature01509 12660783

