# Supplementary material for: Severe Acute Respiratory Syndrome: Temporal Stability and Geographic Variation in Death Rates and Doubling Times
Source: Emerg Infect Dis. 2003 Aug;9(8):991–4. doi: 10.3201/eid0908.030334 (PMC3020622; doi:10.3201/eid0908.030334)
Supplement: Appendix — Severe Acute Respiratory Syndrome: Temporal Stability and Geographic Variation in Case-Fatality Rates and Doubling Times [file 03-0334-app-s1.pdf]

# Appendix

## Definition of Doubling Time

The average doubling time from time $t_0$ to time $t_1$ is simply

$$\frac{t_1 - t_0}{\log_2\left( {N_1}/{N_0} \right)}$$

where $N_1$ and $N_0$ are the number of cases at times $t_1$ and $t_0$, respectively. The units correspond to those used to measure the interval length $t_1$-$t_0$. For example, if $N_1$=2$N_0$, then the average doubling time is exactly $t_1$-$t_0$. In Figures 2 and 3, the time $t_0$ (in days) is always taken to be the earliest time when case counts are available in WHO data.

## Analysis of SARS Data from China

Figure 2 was obtained by (i) first applying a lowess smooth function (9) to the observed case counts, (ii) applying least squares linear fit of the logarithm of the smoothed case counts against time, and (iii) transforming the fitted line back to the original scale. In particular, in (ii) the estimated line is

log(case)=6.2+0.04$t$

(where $t$=time in days, ranging inclusively from the value 1 [March 17] to value 57 [May 12])

## Prediction of Cases with 95% Confidence Interval for March 17, 2003

The predicted confidence interval for $Y_{(new)}$ is the following:

$$\frac{Y_{(new)} - \hat{Y}}{\sqrt{Var(pred)}} \rightarrow t_{(n-2)}, \quad Var(pred) = MSE * \left[ 1 + \frac{1}{n} + \frac{(X_{new} - \overline{X})^2}{\sum (X_i - \overline{X})^2} \right]$$

The estimates and confidence intervals are then transformed back to the original scale.

On March 17, 2003, the estimated case is 502, with a 95% confidence interval (468 to 538); note that this confidence interval is for the actual number of cases on March 17.
